# Respiratory Tract Epithelial Cells Express Retinaldehyde Dehydrogenase ALDH1A and Enhance IgA Production by Stimulated B Cells in the Presence of Vitamin A

**DOI:** 10.1371/journal.pone.0086554

**Published:** 2014-01-22

**Authors:** Rajeev Rudraraju, Bart G. Jones, Sherri L. Surman, Robert E. Sealy, Paul G. Thomas, Julia L. Hurwitz

**Affiliations:** 1 Department of Infectious Diseases, St Jude Children’s Research Hospital, Memphis, Tennessee, United States of America; 2 Department of Immunology, St. Jude Children’s Research Hospital, Memphis, Tennessee, United States of America; 3 Department of Microbiology, Immunology and Biochemistry, University of Tennessee Health Science Center, Memphis, Tennessee, United States of America; Johns Hopkins University - Bloomberg School of Public Health, United States of America

## Abstract

Morbidity and mortality due to viral infections are major health concerns, particularly when individuals are vitamin A deficient. Vitamin A deficiency significantly impairs mucosal IgA, a first line of defense against virus at its point of entry. Previous reports have suggested that CD11c^Hi^ dendritic cells (DCs) of the gastrointestinal tract produce retinaldehyde dehydrogenase (ALDH1A), which metabolizes vitamin A precursors to retinoic acid to support normal mucosal immunity. Given that the upper respiratory tract (URT) and gastrointestinal tract share numerous characteristics, we asked if the CD11c^Hi^ DCs of the URT might also express ALDH1A. To address this question, we examined both CD11c^Hi^ test cells and CD11c^Lo/neg^ control cells from nasal tissue. Surprisingly, the CD11c^Lo/neg^ cells expressed more ALDH1A mRNA per cell than did the CD11c^Hi^ cells. Further evaluation of CD11c^Lo/neg^ populations by PCR and staining of respiratory tract sections revealed that epithelial cells were robust producers of both ALDH1A mRNA and protein. Moreover, CD11c^Lo/neg^ cells from nasal tissue (and a homogeneous respiratory tract epithelial cell line) enhanced IgA production by lipopolysaccharide (LPS)-stimulated splenocyte cultures in the presence of the retinoic acid precursor retinol. Within co-cultures, there was increased expression of MCP-1, IL-6, and GM-CSF, the latter two of which were necessary for IgA upregulation. All three cytokines/chemokines were expressed by the LPS-stimulated respiratory tract epithelial cell line in the absence of splenocytes. These data demonstrate the autonomous potential of respiratory tract epithelial cells to support vitamin A-mediated IgA production, and encourage the clinical testing of intranasal vitamin A supplements in vitamin A deficient populations to improve mucosal immune responses toward respiratory tract pathogens and vaccines.

## Introduction

Vitamin A plays an essential role in a variety of biological functions including the development of healthy immune responses [1]–[5], and vitamin A deficiency is a leading cause of death by infection among children worldwide. Vitamin A deficiencies and insufficiencies exist in both developed and developing countries, particularly among premature infants [6]–[10].

Vitamin A is acquired in the diet and can be stored in the liver as retinyl esters or transported through the circulatory system in the form of retinol bound to retinol binding protein [11]. A ubiquitously distributed subfamily of enzymes, the alcohol dehydrogenases, convert retinol to retinaldehyde, but the further conversion of retinaldehyde to retinoic acid, the metabolite most relevant for activation of the immune response, requires a subfamily of aldehyde dehydrogenases (ALDH1A) with restricted tissue and cell distribution [12]. Retinoic acid functions by binding to retinoic acid receptors (RAR) and retinoid X receptors (RXR), which bind to retinoic acid response elements (RARE) and act as ligand-dependent regulators of transcription [13], [14].

ALDH1A expression has been argued to occur primarily within a few cell types in the gut including dendritic cells (DCs), which upon metabolizing retinaldehyde to retinoic acid, can imprint B cells and T cells with homing receptors and enhance IgA production [15], [16]. Based in part on the clear dependence of gut immune responses on vitamin A, the WHO recommends vitamin A supplementation in vitamin A deficient (VAD) populations at the time of polio virus vaccinations [17].

Given that there are numerous shared features between upper respiratory tract (URT) and gut mucosa, we previously asked if VAD animals would exhibit impaired immune responses of the respiratory tract [18], [19]. Our experiments showed that VAD animals suffered a number of immune abnormalities including reduced frequencies of virus-specific IgA antibody forming cells (AFCs) in the URT and reduced titers of virus-specific IgA in nasal secretions. Given these effects, and with attention to the design of future therapies for vitamin A deficiency, we questioned whether the URT, like the gut, has autonomous potential to metabolize vitamin A and enhance IgA antibody responses.

Because previous literature had focused on the CD11c^Hi^ DCs of the gut as the prominent producers of ALDH1A, we were surprised to find that CD11c^Lo/neg^ cells from the URT expressed more ALDH1A mRNA per cell than did CD11c^Hi^ cells. Dissection of the CD11c^Lo/neg^ population showed that the URT epithelial cells were positive for mRNA expression and that epithelial cells of both URT and lower respiratory tract (LRT) tissues expressed robust levels of ALDH1A protein. We further showed that in the presence of vitamin A precursors, co-cultures with stimulated splenocytes and respiratory tract epithelial cells up-regulated IL-6, GM-CSF, MCP-1 and IgA.

## Materials and Methods

### Ethics Statement

All animal research was conducted in strict accordance with recommendations outlined in the guide for the care and use of laboratory animals of the National Research Council. Experiments were approved by the St. Jude IACUC (protocol #111). Every effort was made to minimize animal suffering.

### Cell Lines

Cell lines included splenic macrophage lines MAC INF-4.2.9 and LIE 13–14, kindly provided by Dr. W.S. Walker [20] and LET1 (LET) cells, a homogeneous T1α-positive C57BL/6 murine respiratory tract lung epithelial cell line immortalized with a retrovirus expressing the SV40 large T antigen (kindly provided by Dr. Carrie M. Rosenberger, Genentech).

### Isolation of URT Nasal Tissue Cells

C57BL/6 mice (Jackson Laboratories, Bar Harbor, Maine) were anesthetized and then exsanguinated by clipping the axillary/brachial artery. Animals were then perfused with 10 ml of PBS to remove remaining blood. Cells were harvested by first detaching heads and removing skin, lower jaws, palates, muscles, cheek bones and incisors. Remaining snouts including both respiratory and olfactory regions were broken into pieces using a syringe plunger. They were then digested with collagenase type II (Worthington Biochemical Corp, Cat# LS004177, 200 U/ml), dispase (Becton Dickinson and Company, Cat# 354235, 50 U/ml) and DNase (Sigma-Aldrich, Cat# D4513,15 U/ml) for 1 hour at 37°C in a shaker at 225 rpm. Cells were passed through a 100 µm cell strainer and washed 2× with complete medium (CM), a Modified Eagles Medium (Invitrogen, Grand Island, NY) supplemented with dextrose (500 µg/ml), glutamine (2 mM), 2-mercaptoethanol (3×10^−5^ M), essential and non-essential amino acids, sodium pyruvate, sodium bicarbonate and antibiotics [21], plus 10% heat inactivated fetal bovine serum (FBS). Dead cells were removed from cell populations with a Ficoll gradient (MP Biomedicals, LLC, Cat# 50494) and Dead Cell Removal beads (Miltenyi Biotech, Cat# 130-090-101). URT cells isolated from nasal tissue by collagenase, dispase and DNase digestions are termed ‘NT’ in this report.

### Separation of CD11c^Hi^, CD11c^Lo/neg^, and Macrophage Populations

CD11c^Hi^ and CD11c^Lo/neg^ cells were separated from NT cells, mesenteric lymph nodes (MesLN), and cervical lymph nodes (CLN) using CD11c microbeads (Miltenyi Biotech, Cat# 130-052-001) per manufacturer’s recommendations. Briefly, cell suspensions were incubated with 100 µl of the CD11c microbeads at 4–8°C for 15 min, washed, and passed through an MS column on a magnet. The bound cells contained the CD11c^Hi^ cells. The effluent was passed through another column on the magnet for maximal depletion of CD11c^Hi^ cells and the remaining cells were collected as the CD11c^Lo/neg^ population. FACS analyses were used to confirm phenotypes. Briefly, cell populations were stained with anti-CD11c antibody (PE-labeled antibody, Miltenyi) and 7-AAD dye for analysis on a FACS Calibur. Live cells and non-RBC were first gated on the basis of forward scatter and exclusion of 7-AAD dye. Histograms were then plotted for anti-CD11c antibody staining (CD11c^Hi^ and CD11c^Lo/neg^ populations) using FlowJo software. Histograms of the CD11c^Hi^ (red) and CD11c^Lo/neg^ (blue) NT cell populations and of control naïve splenocytes (green) are shown in Figure S1. Isolation of macrophages from NT cells was by percoll gradient separation followed by the sorting of live singlets for the phenotype F4/80^+^ (PE anti-F4/80 Biolegend Cat#122616), CD11c^Neg^ (PerCP Cy5.5-anti-CD11c BD Cat#560584), and CD11b^+^ (APC-anti CD11b BD Cat#553312) on a BD FACS Aria II sorter (>99% purity).

### Enrichment of Epithelial Cells from URT or Lung by Negative Selection and Short Term Culture

To enrich epithelial cells for PCR analyses, URT and lung tissues were disrupted and digested with 200 U/ml collagenase type II, 50 U/ml dispase, and 15 U/ml DNase. The mixture was then passed through a 100 µm cell strainer, washed, and plated in a cell culture T75 flask (Corning, Cat# 430641). After 1 hour at 37°C, non-adherent cells were collected and adherent cells were discarded. B and T cells were removed using Dynabeads Mouse Pan B (Invitrogen, Cat#114.41D) and Pan T (Invitrogen, Cat# 114.43D) per the manufacturer’s recommendations. The remaining cells were then plated at a density of 4×10^5^ cells/chamber (2 cm^2^ chambers, Lab Tek II 4 chamber slides, NUNC Cat# 155382), allowed to adhere during a 2 day culture at 37°C in CM, gently washed to remove non-adherent cells, and cultured for 2 additional days in CM.

### PCR Analysis for ALDH1A mRNA

Cells were stored in RNA Protect Cell Reagent (Qiagen, Cat#76526) at −20°C and RNA was extracted from 1×10^5^ cells using RNeasy Plus Mini Kits (Qiagen, Cat# 74134). One third of the extracted RNA was used for cDNA synthesis using Superscript III (Invitrogen, Cat#18080-044). One µl of the neat or 1∶10 serially diluted cDNA (of 40 µl total) was used for PCR amplification with Platinum PCR SuperMix (Invitrogen, Cat#11306-016). 1.2% agarose E-Gels (Invitrogen, Cat# G501801) were run with 5 µl of the PCR product, 2 µl of 10× Blue Juice (Invitrogen Cat# 10816-015) and 13 µl of dH_2_O. Stratagene Eagle Eye II was used to visualize the E-Gels. The primers used in the analyses were: ALDH1A1 forward: 5′-ATGGTTTAGCAGCAGGACTCTTC-3′; ALDH1A1 reverse: 5′-CCAGACATCTTGAATCCACCGAA-3′; ALDH1A2 forward: 5′- GACTTGTAGCAGCTGTCTTCACT-3′; ALDH1A2 reverse: 5′-TCACCCATTTCTCTCCCATTTCC -3′; ALDH1A3 forward: 5′-GGACAGTCTGGATCAACTGCTAC-3′; ALDH1A3 reverse: 5′- TCAGGGGTTCTTCTCCTCGAG T-3′; GAPDH-forward: 5′-CCAGGTTGTCTCCTGCGACTT-3′; GAPDH reverse: 5′-CCTGTTGCTGTAGCCGTATTCA-3′.

### Co-culturing Splenocytes or Mixed, Purified B/T Cells with NT Cells or LET Cells

Spleen cells (4×10^5^ cells per well) or a mixed population of purified B/T cells (2×10^5^ B cells and 2×10^5^ T cells per well) were incubated with 2×10^4^ CD11c^Hi^ or CD11c^Lo/neg^ NT cells or 1×10^3^ LET cells in a 96 well flat bottom plate and stimulated with 1 ug/ml LPS with or without 1 uM retinol (Sigma-Aldrich, Co, Cat# R7632) in a final volume of 200 ul CM per well [14]. Plates were incubated at 37°C for 7 days.

For cytokine neutralization experiments, antibodies were added at the initiation of co-cultures. Anti-GM-CSF was used at 10 µg/ml as recommended by the manufacturer or 20 µg/ml (BD Pharmingen, Cat#554403) and anti-IL-6 was used at 30 ng/ml as recommended by the manufacturer or 150 ng/ml (R&D Systems, Cat#AF406NA).

B and T cells were purified per the manufacturers’ recommendations (Invitrogen, Dynabeads Mouse CD43 [Untouched B cells], Cat# 11422D and Miltenyi, Pan T isolation Kit II, Cat# 130-095-130). Briefly, for T cell purification, 1×10^8^ spleen cells were incubated in 400 µl of buffer and 100 µl of Biotin-antibody cocktail for 10 minutes at 4–8°C. Buffer (300 µl) was added with 200 µl anti-biotin microbeads. Incubation was for 15 minutes at 4–8°C. Cells were washed with 10 ml of buffer, resuspended in 10 ml of buffer and divided into four portions of 2.5 ml, each passed through an individual MS column on a MACs magnetic cell separator. Columns were washed 3× with 2.5 ml of buffer. The effluent contained the enriched T cells. For B cell purification, 1×10^8^ spleen cells in 2 ml of isolation buffer were mixed with 250 µl of pre-washed Dynabeads Mouse CD43 and incubated at room temperature (RT) for 20 minutes on a rotating and tilting platform. Cells were mixed prior to addition of 4 ml isolation buffer and then placed on the magnet for 2 minutes. The medium containing the unbound cells was transferred to an empty tube and once again placed on the magnet to remove any remaining non-B cells.

### Depletion of Antigen Presenting Cells (APC) from Splenocyte Populations

APC were depleted using CD11c (Miltenyi, Cat# 130-050-001) and CD11b (Miltenyi, Cat# 130-049-601) microbeads. Briefly, CD11c+ cells were depleted by incubating 1×10^8^ spleen cells in 400 µl of buffer with100 µl of CD11c microbeads for 15 minutes at 4–8°C. Cells were washed with 15 ml of buffer, resuspended in 500 ul buffer and placed on a pre-rinsed MS column on a MACs separator. The column was washed on the magnet 3× with 500 µl buffer and the effluent containing the CD11c^+^ cell-depleted population was collected. For depletion of the CD11b^+^ population, cells in 900 µl of buffer were incubated with 100 µl of CD11b microbeads for 15 minutes at 4–8°C. Cells were washed with 15 ml of buffer, resuspended in 500 µl of buffer, and placed on a pre-rinsed MS column on a MACs separator. Again, the column was washed on the magnet 3× with 500 µl buffer and the effluent containing the CD11b^+^ cell-depleted population was collected.

### IgA ELISA

Culture supernatants were tested for IgA antibodies by ELISA. 96 well EIA/RIA plates (Corning, Inc, Cat# 9018) were coated with 50 µl of a 1∶1000 dilution of goat anti-mouse IgA-UNLB (Southern Biotech, Cat# 1040-01) and incubated for 4 hours at 37°C. Plates were washed 4× with PBS and blocked overnight at 4°C with 200 µl of PBS containing 2% BSA (Sigma Aldrich, Cat#A8412). Blocking buffer was removed and 50 µl of a 1∶4 dilution of the samples were added to the wells. Mouse IgA (Southern Biotech, Cat# 0106-01) was used as a standard. Plates were incubated at 37°C for 2 hours. Plates were washed 6× with PBS-Tween 20 (0.05%) and incubated with a 1∶1000 dilution of alkaline phosphatase-conjugated goat anti-mouse IgA (Southern Biotech, Cat# 1040-04) in PBS containing 1% BSA and 0.1% Tween 20 for 1 hour at 37°C. Plates were washed 6× with PBS-Tween 20 (0.05%) and developed by addition of p-nitrophenyl phosphate substrate (1 mg/ml) in diethanolamine buffer (0.1 M TRIS, 0.1 M NaCl, 5% Diethanolamine, 10 mM MgCl_2_, pH9.8). The assays were read at OD 405 nm (E max precision microplate reader, Molecular Devices, Inc, Sunnyvale, CA).

### Immunofluorescence Assays

Snouts from naïve mice were fixed with 10% formalin, decalcified with a mixture of formic acid and formaldehyde, sectioned and embedded in paraffin. Lungs were fixed in 10% formalin and embedded in paraffin. Samples were deparaffinized twice for 10 minutes each with xylene. Sections were hydrated by exposure to decreasing concentrations of ethanol (100%, 95%, 70%, 50%, 30% and 0%) for 3 minutes each. Slides were blocked with PBS containing 7.5% BSA for 1 hour at RT. The slides were boiled gently for 30 minutes in heat induced epitope retrieval buffer containing 20 mM TRIS, 1 mM EDTA and 0.05% Tween 20 at pH 8.6. Slides were allowed to cool and washed with PBS for 10 minutes. They were then blocked with 7.5% BSA-PBS in a humidified chamber at RT and washed for 5 minutes with 2% BSA-PBS. Slides were stained with 400 ul of a 1∶100 dilution of rabbit anti-mouse ALDH1A2 (Sigma Aldrich, Cat# SAB4503487) or control rabbit anti-goat IgG (Invitrogen,Cat# A10537) in 2% BSA-PBS and incubated overnight at 4°C. Slides were washed 2× with PBS and stained with 400 µl of a 1∶100 dilution of Alexa Fluor® 568 donkey anti-rabbit IgG (H+L, Invitrogen, Cat# A10042) in 2% BSA-PBS for 3 hours at RT in a humidified chamber. Slides were counterstained by placing two drops of Prolong Gold anti-fade reagent with DAPI (Invitrogen, Cat#P36935). Images were acquired on a Nikon Eclipse E800 microscope.

### Cytokine Analyses

Cytokine/chemokine levels were measured in 25 µl of supernatants taken after 7-day cultures using Milliplex MAP Kit Mouse Cytokine/Chemokine 96-well plate assays (Millipore Corp, Cat# MPXMCYTO70KPMX13) per the manufacturer’s recommendations. The kit was designed to quantify GM-CSF, IFNγ, IL-1β, IL-2, IL-4, IL-5, IL-6, IL-7, IL-10, IL-12, IL-13, MCP-1 (CCL2) and TNFα. Assays were analyzed using bead-based flow cytometry (Luminex 100/200 System, Luminex Corporation, Austin, TX). TGFβ was tested with a Multi Species TGF-β1 Singleplex Bead Kit (Invitrogen, Cat# LHG0121).

### Statistical Analyses

Statistical comparisons were made with unpaired Student’s T tests using GraphPad Prism Software. Values of p<.05 were considered statistically significant. Experiments were repeated to ensure reproducibility.

## Results

### Expression of ALDH1A mRNA among CD11c^Hi^ and CD11c^Lo/neg^ Cells

Previous literature has suggested that CD11c+ DCs from gut-associated lymphoid tissues (GALT) produce ALDH1A enzymes, metabolize retinaldehyde to retinoic acid, and imprint/activate B and T lymphocytes [15], [16]. This information prompted us to question whether CD11c^Hi^ cells of the URT might also express ALDH1A mRNA. To answer this question, we enriched both CD11c^Hi^ and CD11c^Lo/neg^ populations from NT cells and cervical lymph node (CLN) cells. Mesenteric lymph node (MesLN) cells were used as positive controls [1]. RNA was extracted and cDNA was synthesized using oligo-dT primers. We then performed semi-quantitative PCR with serially diluted cDNA.

**Figure 1:**
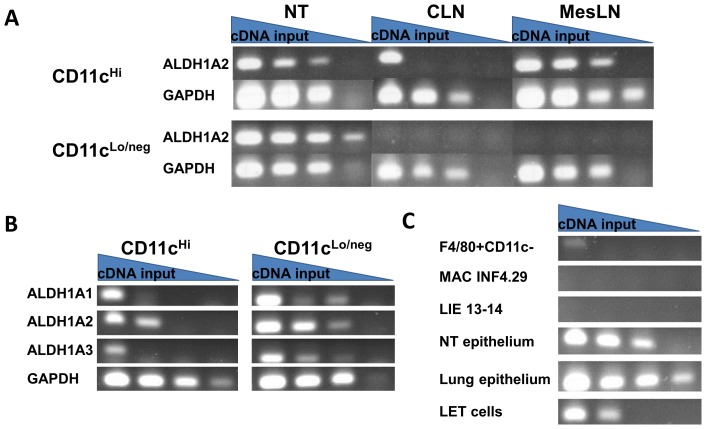
URT cells express ALDH1A mRNA. RNA was extracted from cells and cDNA was synthesized using oligo-dT_20_ primers. Serial 1∶10 dilutions of the cDNA were used for PCR amplifications. Gels were loaded from left to right with PCR products from serially diluted cDNA. The left-most columns were representative of products from ∼1×10^3^ cells. **Panel A.** Results are shown for NT cells (see Materials and Methods), cervical lymph nodes (CLN) and mesenteric lymph nodes (MesLN) of naïve C57BL/6 mice, separated into CD11c^Hi^ and CD11c^Lo/neg^ populations and tested for ALDH1A2 and GAPDH mRNA. **Panel B.** CD11c^Hi^ and CD11c^Lo/neg^ NT populations were tested for ALDH1A1, ALDH1A2, ALDH1A3 and GAPDH mRNA. **Panel C.** Cells were tested for ALDH1A2 mRNA. Samples included NT cells that had been FACS-sorted for the F4/80^+^CD11c^-^CD11b^+^ phenotype (abbreviated ‘F4/80+CD11c-’), two macrophage lines MAC INF4.29 and LIE 13–14, NT cells or lung cells enriched for epithelium by negative selection and short-term culture (see Materials and Methods), and LET cells. On a per-cell basis, the highest ALDH1A expression levels were among CD11c^Lo/neg^ cell populations.

As shown in Figure 1A, ALDH1A2 mRNA was present in the CD11c^Hi^ populations from all three tissues. However, unlike the situation for CLN and MesLN, CD11c^Lo/neg^ NT cells were also strongly positive for ALDH1A mRNA. In fact, on a per cell basis, expression amongst CD11c^Lo/neg^ NT cells exceeded that of the CD11c^Hi^ MesLN positive controls. Tests of ALDH1A1, ALDH1A2, and ALDH1A3 (Figure 1B) further showed that on a per cell basis, all three mRNA species were expressed better among CD11c^Lo/neg^ cells than among CD11c^Hi^ cells of NT.

To characterize the CD11c^Lo/neg^ cell type that expressed ALDH1A mRNA, we tested NT populations that were FACS-sorted for macrophages (F4/80^+^CD11c^-^CD11b^+^) along with two splenic macrophage cell lines, MAC INF4.29 and LIE 13–14 (kindly provided by Dr. W.S. Walker [20], [22]). All signals were weak or negative (Figure 1C). We then enriched epithelial cells from NT and lung cells for testing. This was accomplished by isolating NT and lung populations and negatively selecting for rapidly adherent cells, B cells and T cells. The remaining cells were plated for two days in tissue culture, followed by removal of non-adherent cells and an additional two days in tissue culture. As shown in Figure 1C, the primary epithelial cell-enriched NT and lung populations (termed ‘NT epithelium’ and ‘Lung epithelium’ in the figure) each expressed ALDH1A2 mRNA. Finally, we examined a homogenous T1α-positive respiratory tract epithelial cell line (LET cells). As with the epithelial cell-enriched NT and lung populations, ALDH1A2 mRNA expression was high in the respiratory tract epithelial cell line (Figure 1C).

### ALDH1A2 Protein is Constitutively Expressed at High Levels *in vivo* among Epithelial Cells Lining the URT and LRT Airways

**Figure 2:**
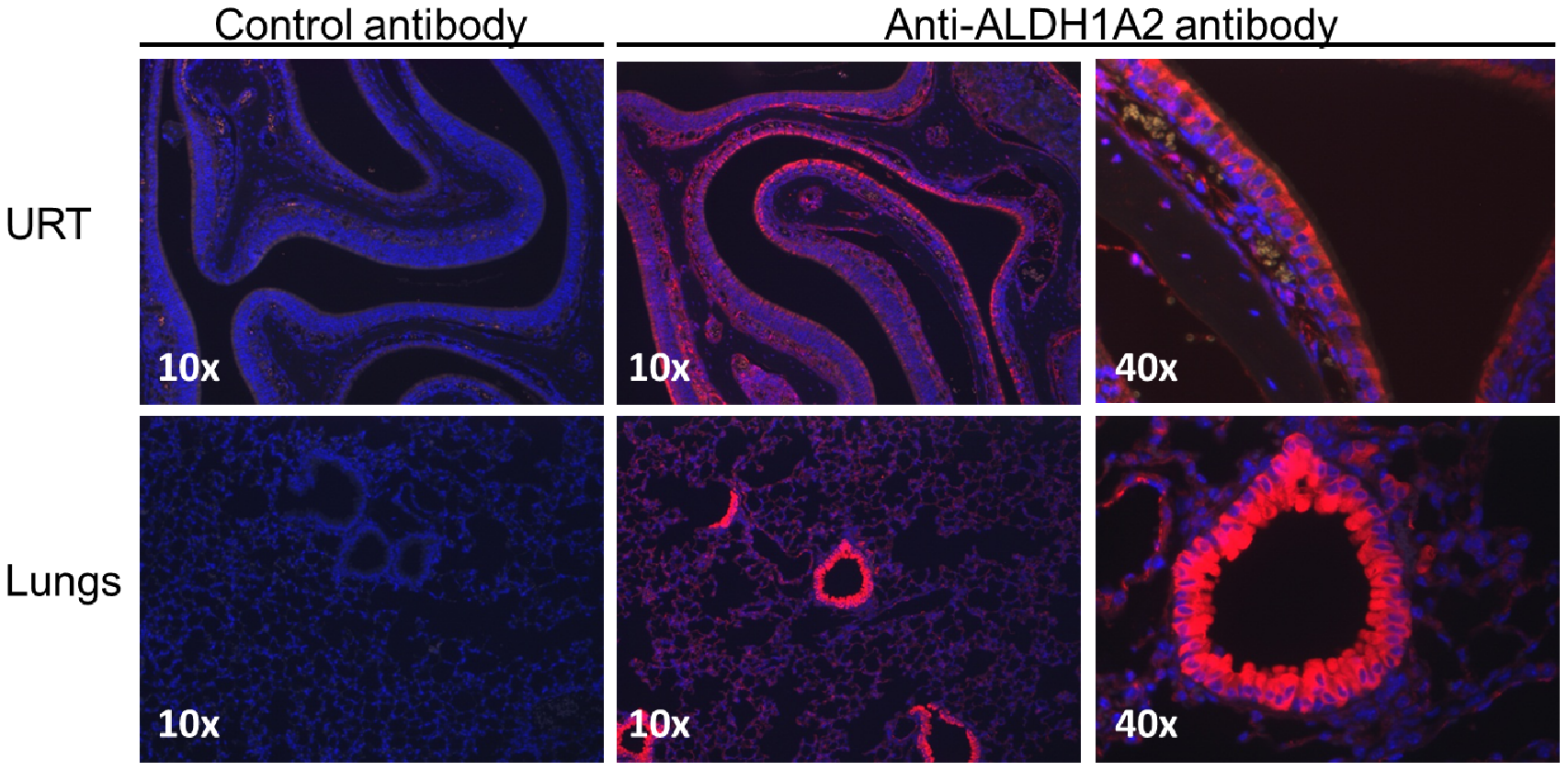
Respiratory tract epithelial cells produce ALDH1A2. Tissue sections were prepared from the URT and lungs of C57BL/6 mice. Sections were stained with rabbit anti-mouse ALDH1A2 or rabbit anti-goat antibody (control). Bound rabbit anti-mouse ALDH1A2 antibody was detected using Alexa Fluor 568 donkey anti-rabbit IgG. The tissues were counter stained with nuclear stain DAPI. Sections of URT and lung are shown at low (10×) and high (40×) magnification. Staining indicated that the greatest ALDH1A2 protein expression was among epithelial cells lining the respiratory tract.

To determine if our detection of ALDH1A mRNA was associated with protein production in the respiratory tract, we performed immunohistochemical analyses on tissue sections of the URT and LRT from naïve C57BL/6 animals (Figure 2). We found that the predominant cells expressing high levels of ALDH1A2 protein in the URT were the epithelial cells lining the airway. We also observed rare positive cells underlying the respiratory tract epithelium, perhaps representative of the CD11c^+^ DC population. In the lung, ALDH1A2 expression was most abundant in the bronchial epithelial cells as compared to alveolar cells. Clearly, ALDH1A expression is not confined to the gut; respiratory tract cells express the enzyme necessary for metabolism of retinaldehyde to retinoic acid.

### CD11c^Lo/neg^ Cells from the URT Enhance IgA Antibody Production in the Presence of Vitamin A

**Figure 3:**
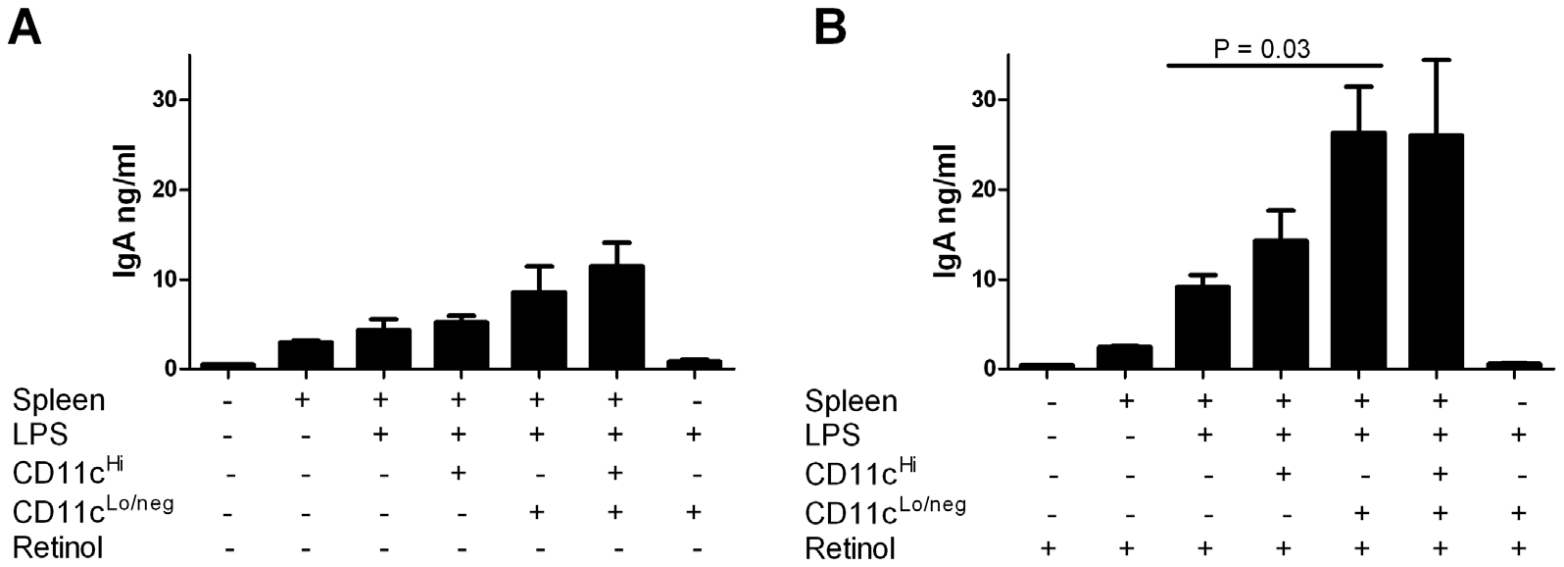
URT cells enhance IgA production in splenocyte co-cultures. Splenocytes were cultured with or without LPS (1 µg/ml) in the absence (**A**) or presence (**B**) of retinol (1 µM). Culture components are indicated below each bar. Where indicated, CD11c^Hi^ and/or CD11c^Lo/neg^ NT cells were added to cultures. IgA was measured (Y axis, ng/ml) from supernatants after a 7 day culture. Comparisons of IgA in cultures with splenocytes, LPS and retinol with or without CD11c^Lo/neg^ NT cells revealed a statistically significant difference (p = 0.03). Comparisons of IgA in cultures with splenocytes, CD11c^Lo/neg^ cells, and LPS with or without retinol also revealed a statistically significant difference (p = 0.04).

Given that URT CD11c^Hi^ and CD11c^Lo/neg^ cells express enzymes necessary for vitamin A metabolism, we asked whether they could also enhance IgA antibody secretion by stimulated B cells in the presence of vitamin A. To this end, we compared IgA levels following a 7 day co-culture of CD11c^Hi^ or CD11c^Lo/neg^ NT cells with LPS-stimulated spleen cells in the absence (Figure 3A) or presence (Figure 3B) of the retinoic acid precursor retinol. As shown in Figure 3, when retinol was added to LPS-stimulated splenocytes in the absence of NT cells, IgA expression increased moderately, suggesting that splenic cells (presumably DCs) have a limited capacity to metabolize vitamin A. The IgA was slightly higher when CD11c^Hi^ NT cells were added to LPS-stimulated splenocytes in the presence of retinol, consistent with the expression of ALDH1A by CD11c^Hi^ NT cells (Figure 1). However, IgA production was highest when CD11c^Lo/neg^ NT cells and retinol were added to stimulated splenocytes [14], [23]. IgA production in these cultures was reproducibly and significantly greater than IgA production in cultures lacking CD11c ^Lo/neg^ NT cells (p = .03) or retinol (p = 0.04).

### A Homogeneous Respiratory Tract Epithelial Cell Line Enhances IgA in Splenocyte Co-cultures

**Figure 4:**
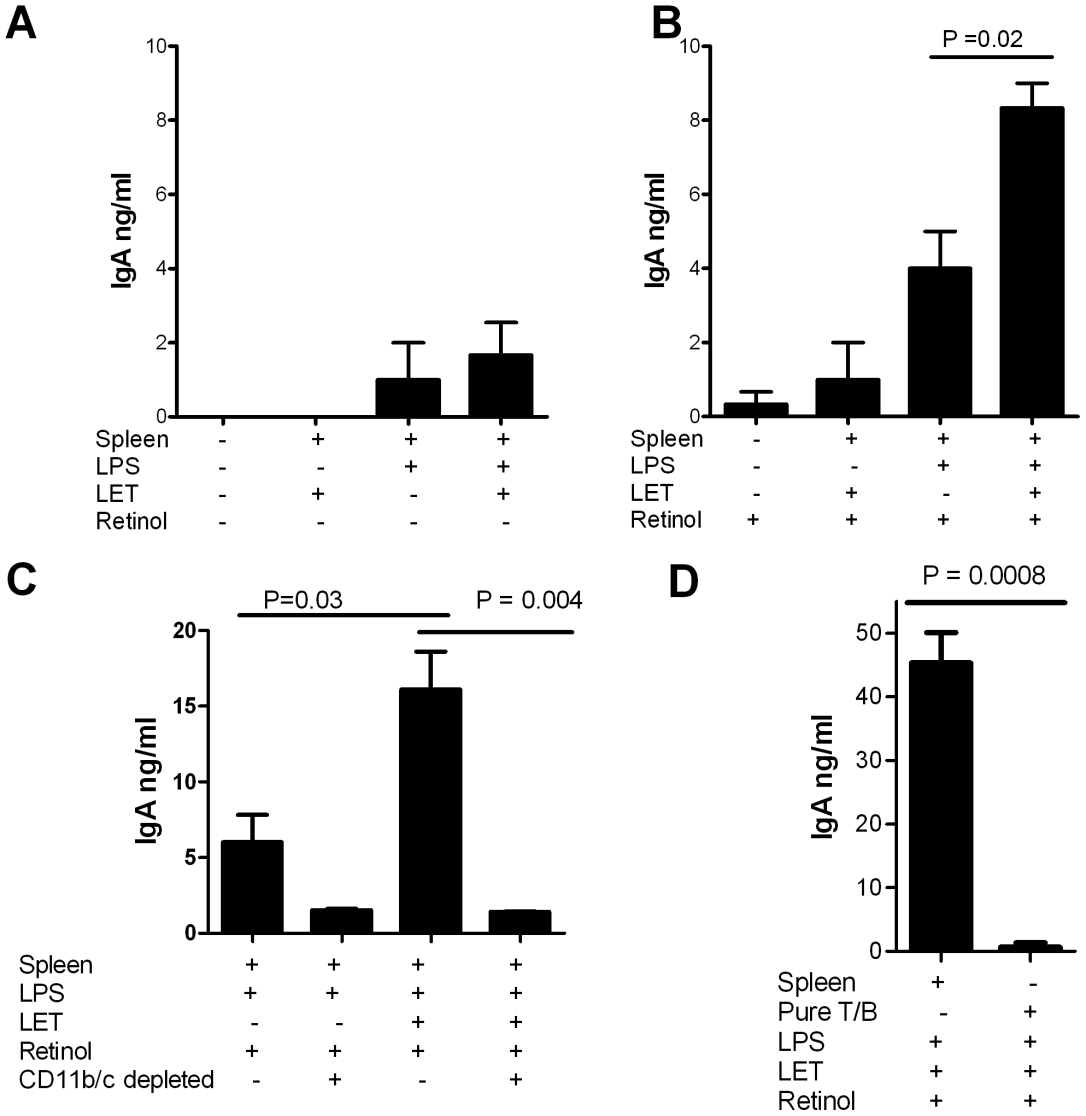
Respiratory tract epithelial cell line enhances IgA production in splenocyte co-cultures. IgA was tested after culture of splenocytes, LPS, and LET cells in various combinations in the absence (**A**) or presence (**B**) of retinol (1 µM). LPS-stimulated splenocytes were also tested in the absence or presence of LET cells, with or without depletion of CD11b^+^/Cd11c^+^ splenocytes (**C**). **Panel D** shows IgA expression in cultures of splenocytes or purified T and B cells in the presence of LPS, LET cells and retinol. Culture components are indicated below each bar.

To confirm that epithelial cells can enhance IgA antibody responses, we replaced the CD11c^Lo/neg^ population with the homogenous respiratory tract epithelial cell line LET. As shown in Figures 4A and B, in the presence of retinol, LET cells significantly improved IgA production by stimulated B cells. A greater increase was induced by the combination of LET cells and retinol than by either LET cells or retinol alone. Nonetheless, we found that LET cells had no effect in the absence of conventional APCs. When CD11b^+^ and CD11c^+^ APCs were depleted, IgA stimulation was lost (Figure 4C). In addition, when purified splenic B cells and T cells were used as a replacement for splenocytes, LET cells were not sufficient to promote IgA production in the presence of LPS and retinol (Figure 4D). These results confirmed that respiratory tract epithelial cells have the capacity to enhance IgA antibody responses by stimulated B cells in the presence of vitamin A, provided that conventional APCs are present.

### Respiratory Tract Epithelial Cells Alter the Cytokine Environment

**Figure 5:**
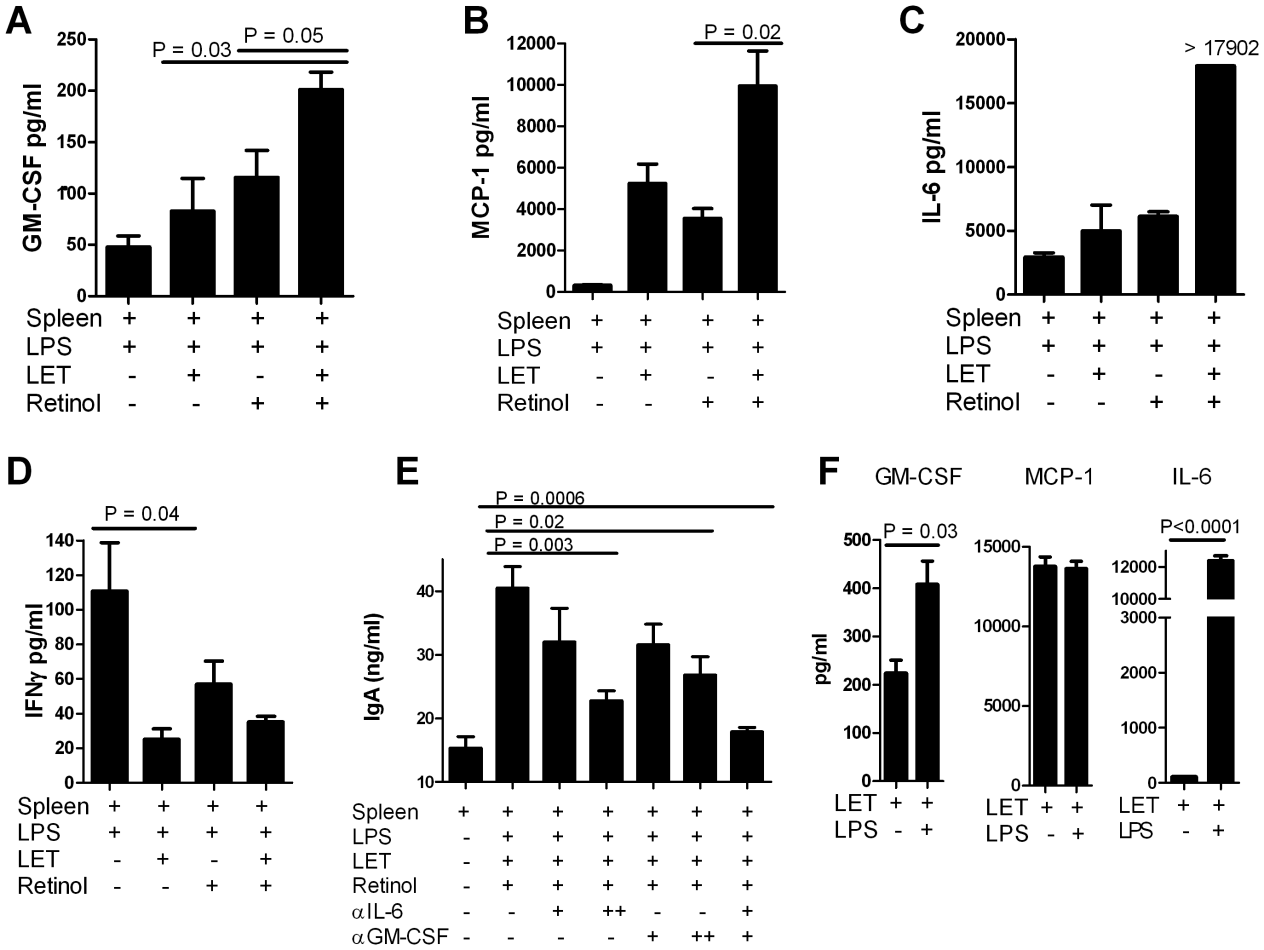
Cytokine production in splenocyte co-cultures. Spleen cells were stimulated with LPS in the presence of LET cells and/or retinol. Culture components are indicated below each bar. Cytokines that were reproducibly elevated in the presence of LET cells and retinol are shown, including GM-CSF (**Panel A**), MCP-1 (**Panel B**), and IL-6 (**Panel C**). IFNγ levels were reduced in the presence of LET cells and retinol (**Panel D**). **Panel E:** IgA levels are shown following splenocyte/LET co-cultures in the presence of LPS and retinol, with and without neutralization using anti-IL-6 and/or anti-GMC-SF antibodies. ‘++’ indicates that antibodies were added to cultures at levels exceeding manufacturers’ recommendations, either 5× (for anti-IL-6 antibodies) or 2× (for anti-GM-CSF antibodies). **Panel F:** Levels of GM-CSF, MCP-1 and IL-6 are shown in LET cell cultures (without splenocytes) in the presence or absence of LPS.

Because IgA up-regulation in the co-culture system was likely mediated, at least in part, by cytokines/chemokines, we examined the levels of GM-CSF, IFNγ, IL-1β, IL-2, IL-4, IL-5, IL-6, IL-7, IL-10, IL-12, IL-13, MCP-1, TNFα, and TGF-β in culture supernatants. In Figure 5A-C are shown results for cytokines that were significantly and reproducibly increased when LET cells and retinol were added to LPS-stimulated splenocyte cultures. Increases were observed in GM-CSF, MCP-1 (CCL2)[24] and IL-6 on day 7 of co-culture. Figure 5D shows that IFNγ was reciprocally reduced when LET cells were added to splenocyte cultures.

GM-CSF is well known for its capacity to activate DCs (and upregulate their retinoic acid–producing capacity [25]), while IL-6 is well known for its capacity to support IgA production by stimulated B cells in the presence of APCs. To determine if these two cytokines were necessary for induction of IgA antibody responses in our co-culture system, we neutralized cytokines with anti-IL-6 and anti-GM-CSF antibodies (Figure 5E). Antibody concentrations were used at concentrations recommended by manufacturers for neutralization and at higher doses (5× for anti-IL-6 antibody and 2× for anti-GM-CSF antibody, indicated as++in Figure 5E). The addition of individual antibodies to cultures, even at levels exceeding those recommended by manufacturers, partially reduced IgA responses. However, when antibodies were mixed together at the recommended doses, IgA responses were reduced to near-background levels. These results demonstrated that IL-6 and GM-CSF jointly contributed to upregulation of IgA antibody responses in the co-culture setting.

Finally, we asked if the homogeneous LET respiratory tract epithelial cell line could directly secrete the upregulated cytokines/chemokines shown in Figures 5A–C. We cultured 10^4^ LET cells/well in CM with and without LPS and tested cultures on day 7 for the cytokines/chemokines GM-CSF, IFNγ, IL-1β, IL-2, IL-4, IL-5, IL-6, IL-7, IL-10, IL-12, IL-13, MCP-1, TNFα, and TGFβ. In the presence of LETs and LPS, but no other cell types, cytokines GM-CSF, MCP-1 and IL-6 were expressed at levels >150 pg/ml (Figure 5F). Both GM-CSF and IL-6 production were increased by LPS stimulation, whereas MCP-1 expression was constitutive. Clearly, the respiratory tract epithelial cells bore receptor molecules supportive of LPS activation in the culture system [26]–[28]. Cytokine/chemokine secretion did not require retinol in these cultures. Cytokines that were not reproducibly detected in LET cultures included IFNγ, IL-1β, IL-2, IL-4, IL-7, IL-10, and TNFα. The cytokine IL-5 was detected above background at low levels and was comparable in cultures with and without LPS. Cytokines IL-12 and IL-13 showed reproducible, but only mild increases in LPS-stimulated cultures. TGFβ levels (tested with a separate ELISA) did not exceed those of medium alone in LET cultures. Results for 13 cytokines/chemokines are shown in Table 1. These results show that the respiratory tract epithelial cells are independently capable of cytokine secretion and may contribute both directly and indirectly to the changes observed in the co-culture system (Figure 5A–C).

**Table 1:** Upregulation of cytokines/chemokines by stimulation of respiratory tract epithelial cells*.

Cytokine/Chemokine	Medium only	LET	LET+LPS
GM-CSF	<22.0	**565**	**768**
IFNγ	<2.4	<2.4	<2.4
IL-1β	<1.6	<1.6	<1.6
IL-2	<3.0	<3.0	<3.0
IL-4	<2.9	<2.9	<2.9
IL-5	<1.9	10.2	11.3
IL-6	<3.2	**1,264**	**10,812**
IL-7	<3.1	<3.1	<3.1
IL-10	<2.7	<2.7	<2.7
IL-12	<2.1	<2.1	27.3
IL-13	<2.1	<2.1	104
MCP-1	<2.5	**>13,912**	**>13,912**
TNFα	<2.6	<2.6	<2.6

* LET cells (1×10^4^ cells per well) were cultured with or without LPS. A sample of culture supernatant (25 µl) was tested using bead-based flow cytometry to compare values (pg/ml). Values exceeding 150 pg/ml are bolded.

## Discussion

We previously showed that vitamin A is critical for the normal development of virus-specific immune responses in the respiratory tract mucosa. Specifically, in a VAD mouse model, we demonstrated that both IgA antibody responses and CD8+ T cell responses toward an intranasal viral infection were impaired [18], [19]. With a goal of correcting the defect, we sought to understand the potentials of URT cells and to test the hypothesis that URT cells can autonomously express ALDH1A. The possibility had not been previously tested, because previous literature suggested that retinoic acid metabolism was largely attributed to CD11c^Hi^ DCs of the gut, which were also able to imprint homing and activation potentials on B cell and T cell populations [1], [15], [16], [25]. Surprisingly, we found that ALDH1A mRNA is expressed by both CD11c^Hi^ and CD11c^Lo/neg^ cells of the URT and that ALDH1A protein is strongly expressed by epithelial cells lining the URT and LRT airways. Further, epithelial cells of the respiratory tract are able to up-regulate IgA production by stimulated B cells in the presence of retinol, a precursor of retinoic acid. Results emphasize that vitamin A metabolism is not dependent solely on intestinal cells and highlight shared features between the intestinal and respiratory tract mucosa [29].

### Upper Respiratory Tract Epithelial Cell Potentials

It is well known that epithelial cells lining the respiratory tract provide a formidable barrier to viruses and other pathogens in the airway. Barriers are supported in part by the dense packing of cells and tight junctions between cells, and by the mucus layer, replete with mucins, anti-microbial peptides and inhibitory proteins (C-reactive protein, elastase, defensins, collectins (surfactant), lysozyme, and lactoferrin [30]–[32]). In addition to providing physical barriers, the respiratory tract epithelial cells actively sample viral antigens, secrete cytokines/chemokines, and modulate innate and adaptive immune responses at the site of infection [32]. These processes are initiated when pathogen components bind the epithelial cell’s germ-line encoded pattern recognition receptors (PRRs) [33] tailored for the class of infectious agent [34], [35]. Once epithelial cells respond to pathogen associated molecular patterns (PAMPs), they transduce signals and upregulate cytokines/chemokines [36]–[42] to recruit and activate other immune effectors. Results in the present report add to current knowledge by demonstrating that respiratory tract epithelial cells situated at the pathogen’s point-of-entry have the capacity to (i) express ALDH1A mRNA and proteins and (ii) induce vitamin A-dependent IgA. The quality and quantity of immune functions in the URT are critical to the outcome of infections, as inhibition of pathogen in the URT may prevent pathogen migration to mid- and lower-respiratory tract tissues and thereby prevent serious damage to the trachea and lungs.

### Cytokines, Vitamin A and IgA Upregulation

In our *in vitro* system, cytokine/chemokine secretion was coincident with IgA up-regulation suggesting a cause-effect relationship. Specifically, GM-CSF, MCP-1 and IL-6 levels were enhanced and IFNγ levels were reduced in the presence of respiratory tract epithelial cells and retinol. The downregulation of IFNγ was not surprising as Th1 (IFNγ) and Th2 (IL-6) cytokines are often cross-regulated, due in part to the interplay of STAT1 and STAT3-dependent functions [43].

Inhibition of GM-CSF and IL-6 reduced IgA to near-background levels showing that these two cytokines were necessary for IgA production in our stimulated B cell cultures. We also showed that in the absence of splenocytes, the homogeneous respiratory tract epithelial cell line LET was independently capable of producing all three chemokines/cytokines; MCP-1 was constitutively expressed, whereas GM-CSF and IL-6 increased when LET cells were stimulated with LPS. Therefore, the cytokines/chemokines measured in co-cultures could have been, at least in part, produced directly by the input epithelial cells. Despite their cytokine/chemokine expression capacity, the LET cells were not capable of upregulating IgA in the absence of CD11b^+^ and CD11c^+^ cells. This may have been a consequence of too little cytokine/chemokine production by LET cells alone (note that only 10^3^ cells were added to co-cultures), but APC contributions other than cytokine release [25], [44], [45] were likely essential for B cell activation. Important APC functions include secretion of soluble CD14 to assist LPS-mediated stimulation [27], activation of T cells, and facilitation of cognate B-T cell interactions.

We propose that IgA production by stimulated B cells is the consequence of a complex interplay among lymphocytes, APCs and epithelial cells. These cells rarely act in isolation, but are engaged in perpetual cross-talk. While epithelial cells trigger APCs (as with GM-CSF-driven DC maturation and induction of retinoic acid producing capacity [25]), APCs up-regulate epithelial cell activities. As in the gastrointestinal tract, respiratory tract APCs, stromal cells and lymphocytes must act in unison to define the cytokine/chemokine milieu, upregulate IgA, and modulate other pathogen-specific effector functions [46].

Our findings concerning vitamin A, cytokine production, and IgA up-regulation were consistent with previous reports, although previous studies have focused more directly on macrophages, DCs or T cells [47]–[50]. For example, it was previously shown that retinoic acid can interact with its receptors to enhance Th2 responses (e.g. IL-6) and suppress APC-mediated induction of IFNγ by Th1 cells [51], [52]. Moreover, each of the three cytokines/chemokines MCP-1, GM-CSF and IL-6 have been shown to associate with improved IgA expression [24], [53]–[56]. IL-6 support of the terminal differentiation of B lymphocytes into IgA-secreting cells is particularly well documented [57], [58].

### A Working Hypothesis for Vitamin A-mediated Epithelial Cell Function in the URT

**Figure 6:**
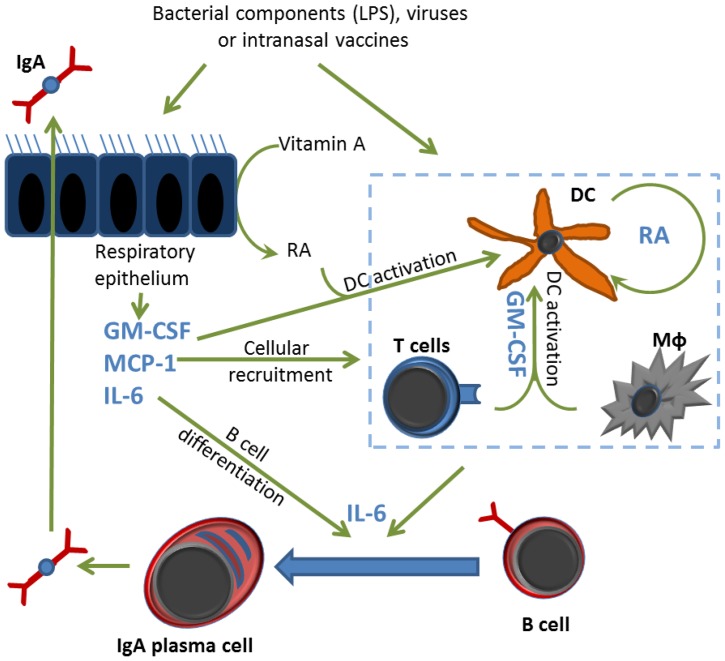
Working hypothesis for epithelial cell-mediated induction of IgA production in the respiratory tract. The cartoon illustrates hypothesized mechanisms of vitamin A-dependent IgA production in the respiratory tract. RA = retinoic acid, Macrophage = MΦ.

Although a number of as yet unidentified factors likely contribute to vitamin A-dependent enhancement of IgA antibody responses *in vivo*, we propose one working hypothesis (illustrated in Figure 6) to explain activities in the URT epithelial cell lining. We propose that respiratory tract epithelial cells with ALDH1A activity are the first cells to react to pathogens and metabolize vitamin A. Their release of MCP-1 and GM-CSF will recruit adaptive and innate immune cells to the site of infection and stimulate DC maturation (leading to increased vitamin A metabolism by DCs)[25]. Cognate B-T cell interactions, facilitated by an abundance of IL-6 (likely secreted by epithelial cells, DCs and T cells) then drive the maturation of B cells to IgA-antibody forming cells, supporting IgA secretion and transcytosis into the URT lumen. This crucial first line of defense may then quell pathogens at their point-of-entry, preventing pathogen descent into mid- and lower respiratory tract tissues, and thereby preventing lung damage. Our working hypothesis may assist the design of future in vivo experiments.

### Clinical Implications

Prompted by our finding that respiratory tract cells produce ALDH1A, we recently asked if intranasal vitamin A supplementation could correct impaired URT production of anti-viral IgA antibodies in the context of vitamin A deficiency. Vitamin supplementation by the intranasal route would be attractive in the clinical arena, as it would (i) deliver vitamin directly to the target tissue and bypass requirements for nutrient transport from the gut, a process that may be impaired in situations of malnutrition or disease, and (ii) allow co-formulation of vaccine with vitamin, simplifying the logistics of any vaccination/vitamin supplementation program. Our pre-clinical tests were successful in that intranasal vitamin A supplementation corrected impaired virus-induced IgA responses in VAD mice (unpublished data).

Vitamin A is essential for normal immune functions [3], [59]–[61], but oral vitamin A supplementation can in some cases cause adverse events in pre-clinical and clinical settings [62]–[64], By applying vitamin A intranasally rather than orally, systemic adverse events may possibly be eliminated. If this suggestion proves to be correct, the intranasal administration of vitamin A may ultimately enhance protection against respiratory pathogens in VAD populations worldwide.

## Supporting Information

Figure S1 **CD11c expression of selected NT cells.** Following enrichment of CD11c^Hi^ and CD11c^Lo/neg^ cells from NT, phenotypes were confirmed by staining populations for membrane CD11c and examination by flow cytometry with a FACS Calibur. Live, non-RBC populations were first gated for analysis. Overlapping histograms represent the enriched CD11c^Hi^ cells (red), CD11c^Lo/neg^ cells (blue), and control naïve splenocytes (green). Relative cell numbers are shown on the Y axis and cell staining intensities are shown on the X axis.
(TIF)

## References

[1] Iwata M, Hirakiyama A, Eshima Y, Kagechika H, Kato C, et al (2004) Retinoic acid imprints gut-homing specificity on T cells. Immunity 21: 527–538. 15485630 10.1016/j.immuni.2004.08.011

[2] Mark M, Ghyselinck NB, Chambon P (2006) Function of retinoid nuclear receptors: lessons from genetic and pharmacological dissections of the retinoic acid signaling pathway during mouse embryogenesis. Annu Rev Pharmacol Toxicol 46: 451–480. 16402912 10.1146/annurev.pharmtox.46.120604.141156

[3] Cassani B, Villablanca EJ, De CJ, Wang S, Mora JR (2012) Vitamin A and immune regulation: role of retinoic acid in gut-associated dendritic cell education, immune protection and tolerance. Mol Aspects Med 33: 63–76. 22120429 10.1016/j.mam.2011.11.001 PMC3246074

[4] Sommer A, Tarwotjo I, Hussaini G, Susanto D (1983) Increased mortality in children with mild vitamin A deficiency. Lancet 2: 585–588. 6136744 10.1016/s0140-6736(83)90677-3

[5] Sommer A, Katz J, Tarwotjo I (1984) Increased risk of respiratory disease and diarrhea in children with preexisting mild vitamin A deficiency. Am J Clin Nutr 40: 1090–1095. 6496388 10.1093/ajcn/40.5.1090

[6] Stephens D, Jackson PL, Gutierrez Y (1996) Subclinical vitamin A deficiency: a potentially unrecognized problem in the United States. Pediatr Nurs 22: 377–89, 456. 9087069

[7] Njuguna M, Msukwa G, Shilio B, Tumwesigye C, Courtright P, et al (2009) Causes of severe visual impairment and blindness in children in schools for the blind in eastern Africa: changes in the last 14 years. Ophthalmic Epidemiol 16: 151–155. 19437309 10.1080/09286580902738183

[8] Mora JR, Iwata M, Von Andrian UH (2008) Vitamin effects on the immune system: vitamins A and D take centre stage. Nat Rev Immunol 8: 685–698. 19172691 10.1038/nri2378 PMC2906676

[9] Shenai JP, Chytil F, Jhaveri A, Stahlman MT (1981) Plasma vitamin A and retinol-binding protein in premature and term neonates. J Pediatr 99: 302–305. 7195934 10.1016/s0022-3476(81)80484-2

[10] Schleicher RL, Sternberg MR, Pfeiffer CM (2013) Race-ethnicity is a strong correlate of circulating fat-soluble nutrient concentrations in a representative sample of the U.S. Population. J Nutr 143: 966S–976S. 23596163 10.3945/jn.112.172965 PMC4802853

[11] Napoli JL (2012) Physiological insights into all-trans-retinoic acid biosynthesis. Biochim Biophys Acta 1821: 152–167. 21621639 10.1016/j.bbalip.2011.05.004 PMC3179567

[12] Mora JR, Von Andrian UH (2009) Role of retinoic acid in the imprinting of gut-homing IgA-secreting cells. Semin Immunol 21: 28–35. 18804386 10.1016/j.smim.2008.08.002 PMC2663412

[13] Samarut E, Rochette-Egly C (2012) Nuclear retinoic acid receptors: conductors of the retinoic acid symphony during development. Mol Cell Endocrinol 348: 348–360. 21504779 10.1016/j.mce.2011.03.025

[14] Tokuyama H, Tokuyama Y (1993) Retinoids enhance IgA production by lipopolysaccharide-stimulated murine spleen cells. Cell Immunol 150: 353–363. 8370078 10.1006/cimm.1993.1203

[15] Mora JR, Iwata M, Eksteen B, Song SY, Junt T, et al (2006) Generation of gut-homing IgA-secreting B cells by intestinal dendritic cells. Science 314: 1157–1160. 17110582 10.1126/science.1132742

[16] Mora JR, Bono MR, Manjunath N, Weninger W, Cavanagh LL, et al (2003) Selective imprinting of gut-homing T cells by Peyer’s patch dendritic cells. Nature 424: 88–93. 12840763 10.1038/nature01726

[17] Bahl R, Bhandari N, Kant S, Molbak K, Ostergaard E, et al (2002) Effect of vitamin A administered at Expanded Program on Immunization contacts on antibody response to oral polio vaccine. Eur J Clin Nutr 56: 321–325. 11965508 10.1038/sj.ejcn.1601325

[18] Rudraraju R, Surman SL, Jones BG, Sealy R, Woodland DL, et al (2012) Reduced frequencies and heightened CD103 expression among virus-induced CD8(+) T cells in the respiratory tract airways of vitamin A-deficient mice. Clin Vaccine Immunol 19: 757–765. 22398245 10.1128/CVI.05576-11 PMC3346339

[19] Surman SL, Rudraraju R, Sealy R, Jones B, Hurwitz JL (2012) Vitamin A deficiency disrupts vaccine-induced antibody-forming cells and the balance of IgA/IgG isotypes in the upper and lower respiratory tract. Viral Immunol 25: 341–344. 22813425 10.1089/vim.2012.0023 PMC3413069

[20] Wilson CM, Gatewood JW, McCormack JM, Walker WS (1991) Immortalization of growth factor-dependent mouse splenic macrophages derived from cloned progenitors. J Immunol Methods 137: 17–25. 1707081 10.1016/0022-1759(91)90389-w

[21] Kappler JW, Skidmore B, White J, Marrack P (1981) Antigen-inducible, H-2-restricted, interleukin-2-producing T cell hybridomas. Lack of independent antigen and H-2 recognition. J Exp Med 153: 1198–1214. 6166712 10.1084/jem.153.5.1198 PMC2186156

[22] McCormack JM, Askew D, Walker WS (1993) Alloantigen presentation by individual clones of mouse splenic macrophages. Selective expression of IL-1 alpha in response to CD8+ T cell-derived IFN-gamma defines the alloantigen-presenting phenotype. J Immunol 151: 5218–5227. 8228220

[23] Tokuyama Y, Tokuyama H (1996) Retinoids as Ig isotype-switch modulators. The role of retinoids in directing isotype switching to IgA and IgG1 (IgE) in association with IL-4 and IL-5. Cell Immunol 170: 230–234. 10.1006/cimm.1996.0156 8674128

[24] Xu LL, Warren MK, Rose WL, Gong W, Wang JM (1996) Human recombinant monocyte chemotactic protein and other C-C chemokines bind and induce directional migration of dendritic cells in vitro. J Leukoc Biol 60: 365–371. 8830793 10.1002/jlb.60.3.365

[25] Yokota A, Takeuchi H, Maeda N, Ohoka Y, Kato C, et al (2009) GM-CSF and IL-4 synergistically trigger dendritic cells to acquire retinoic acid-producing capacity. Int Immunol 21: 361–377. 19190084 10.1093/intimm/dxp003 PMC2660862

[26] Ortega-Cava CF, Ishihara S, Rumi MA, Kawashima K, Ishimura N, et al (2003) Strategic compartmentalization of Toll-like receptor 4 in the mouse gut. J Immunol 170: 3977–3985. 12682225 10.4049/jimmunol.170.8.3977

[27] Jersmann HP (2005) Time to abandon dogma: CD14 is expressed by non-myeloid lineage cells. Immunol Cell Biol 83: 462–467. 16174094 10.1111/j.1440-1711.2005.01370.x

[28] Pugin J, Schurer-Maly CC, Leturcq D, Moriarty A, Ulevitch RJ, et al (1993) Lipopolysaccharide activation of human endothelial and epithelial cells is mediated by lipopolysaccharide-binding protein and soluble CD14. Proc Natl Acad Sci U S A 90: 2744–2748. 7681988 10.1073/pnas.90.7.2744 PMC46172

[29] Rudraraju R, Surman S, Jones B, Sealy R, Woodland DL, et al (2011) Phenotypes and functions of persistent Sendai virus-induced antibody forming cells and CD8+ T cells in diffuse nasal-associated lymphoid tissue typify lymphocyte responses of the gut. Virology 410: 429–436. 21227475 10.1016/j.virol.2010.12.017 PMC3941175

[30] Tripathi S, Tecle T, Verma A, Crouch E, White M, et al (2013) The human cathelicidin LL-37 inhibits influenza A viruses through a mechanism distinct from that of surfactant protein D or defensins. J Gen Virol 94: 40–49. 23052388 10.1099/vir.0.045013-0 PMC3542722

[31] Doss M, White MR, Tecle T, Gantz D, Crouch EC, et al (2009) Interactions of alpha-, beta-, and theta-defensins with influenza A virus and surfactant protein D. J Immunol. 182: 7878–7887. 10.4049/jimmunol.0804049 19494312

[32] Proud D, Leigh R (2011) Epithelial cells and airway diseases. Immunol Rev 242: 186–204. 21682746 10.1111/j.1600-065X.2011.01033.x

[33] Wang Y, Devkota S, Musch MW, Jabri B, Nagler C, et al (2010) Regional mucosa-associated microbiota determine physiological expression of TLR2 and TLR4 in murine colon. PLOS ONE 5: e13607. 21042588 10.1371/journal.pone.0013607 PMC2962643

[34] Kanneganti TD (2010) Central roles of NLRs and inflammasomes in viral infection. Nat Rev Immunol 10: 688–698. 20847744 10.1038/nri2851 PMC3909537

[35] Zaki MH, Boyd KL, Vogel P, Kastan MB, Lamkanfi M, et al (2010) The NLRP3 inflammasome protects against loss of epithelial integrity and mortality during experimental colitis. Immunity 32: 379–391. 20303296 10.1016/j.immuni.2010.03.003 PMC2982187

[36] Willart MA, Deswarte K, Pouliot P, Braun H, Beyaert R, et al (2012) Interleukin-1alpha controls allergic sensitization to inhaled house dust mite via the epithelial release of GM-CSF and IL-33. J Exp Med 209: 1505–1517. 22802353 10.1084/jem.20112691 PMC3409497

[37] Kumar H, Kawai T, Akira S (2011) Pathogen recognition by the innate immune system. Int Rev Immunol 30: 16–34. 21235323 10.3109/08830185.2010.529976

[38] Franchi L, Munoz-Planillo R, Nunez G (2012) Sensing and reacting to microbes through the inflammasomes. Nat Immunol 13: 325–332. 22430785 10.1038/ni.2231 PMC3449002

[39] Hancock RE, Nijnik A, Philpott DJ (2012) Modulating immunity as a therapy for bacterial infections. Nat Rev Microbiol 10: 243–254. 22421877 10.1038/nrmicro2745

[40] Abreu MT (2010) Toll-like receptor signalling in the intestinal epithelium: how bacterial recognition shapes intestinal function. Nat Rev Immunol 10: 131–144. 20098461 10.1038/nri2707

[41] Brandl K, Sun L, Neppl C, Siggs OM, Le Gall SM, et al (2010) MyD88 signaling in nonhematopoietic cells protects mice against induced colitis by regulating specific EGF receptor ligands. Proc Natl Acad Sci U S A 107: 19967–19972. 21041656 10.1073/pnas.1014669107 PMC2993336

[42] Shaykhiev R, Behr J, Bals R (2008) Microbial patterns signaling via Toll-like receptors 2 and 5 contribute to epithelial repair, growth and survival. PLOS ONE 3: e1393. 18167552 10.1371/journal.pone.0001393 PMC2148109

[43] Costa-Pereira AP, Tininini S, Strobl B, Alonzi T, Schlaak JF, et al (2002) Mutational switch of an IL-6 response to an interferon-gamma-like response. Proc Natl Acad Sci U S A 99: 8043–8047. 12060750 10.1073/pnas.122236099 PMC123017

[44] Van Amersfoort ES, Van Berkel TJ, Kuiper J (2003) Receptors, mediators, and mechanisms involved in bacterial sepsis and septic shock. Clin Microbiol Rev 16: 379–414. 12857774 10.1128/CMR.16.3.379-414.2003 PMC164216

[45] Patke CL, Shearer WT (2000) gp120- and TNF-alpha-induced modulation of human B cell function: proliferation, cyclic AMP generation, Ig production, and B-cell receptor expression. J Allergy Clin Immunol 105: 975–982. 10808179 10.1067/mai.2000.105315

[46] Rumbo M, Anderle P, Didierlaurent A, Sierro F, Debard N, et al (2004) How the gut links innate and adaptive immunity. Ann N Y Acad Sci 1029: 16–21. 15681739 10.1196/annals.1309.003

[47] Churchill L, Friedman B, Schleimer RP, Proud D (1992) Production of granulocyte-macrophage colony-stimulating factor by cultured human tracheal epithelial cells. Immunology 75: 189–195. 1537596 PMC1384823

[48] Cromwell O, Hamid Q, Corrigan CJ, Barkans J, Meng Q, et al (1992) Expression and generation of interleukin-8, IL-6 and granulocyte-macrophage colony-stimulating factor by bronchial epithelial cells and enhancement by IL-1 beta and tumour necrosis factor-alpha. Immunology 77: 330–337. 1478679 PMC1421719

[49] Roy RM, Wuthrich M, Klein BS (2012) Chitin elicits CCL2 from airway epithelial cells and induces CCR2-dependent innate allergic inflammation in the lung. J Immunol 189: 2545–2552. 22851704 10.4049/jimmunol.1200689 PMC3424300

[50] Suzuki Y, Suda T, Furuhashi K, Shibata K, Hashimoto D, et al (2012) Mouse CD11bhigh lung dendritic cells have more potent capability to induce IgA than CD103+ lung dendritic cells in vitro. Am J Respir Cell Mol Biol 46: 773–780. 22268142 10.1165/rcmb.2011-0329OC

[51] Iwata M, Eshima Y, Kagechika H (2003) Retinoic acids exert direct effects on T cells to suppress Th1 development and enhance Th2 development via retinoic acid receptors. Int Immunol 15: 1017–1025. 12882839 10.1093/intimm/dxg101

[52] Cantorna MT, Nashold FE, Chun TY, Hayes CE (1996) Vitamin A down-regulation of IFN-gamma synthesis in cloned mouse Th1 lymphocytes depends on the CD28 costimulatory pathway. J Immunol 156: 2674–2679. 8609382

[53] Panee J (2012) Monocyte Chemoattractant Protein 1 (MCP-1) in obesity and diabetes. Cytokine 60: 1–12. 22766373 10.1016/j.cyto.2012.06.018 PMC3437929

[54] Struyf S, Van CE, Paemen L, Put W, Lenaerts JP, et al (1998) Synergistic induction of MCP-1 and -2 by IL-1beta and interferons in fibroblasts and epithelial cells. J Leukoc Biol 63: 364–372. 9500525 10.1002/jlb.63.3.364

[55] Loudon PT, Yager EJ, Lynch DT, Narendran A, Stagnar C, et al (2010) GM-CSF increases mucosal and systemic immunogenicity of an H1N1 influenza DNA vaccine administered into the epidermis of non-human primates. PLOS One 5: e11021. 20544035 10.1371/journal.pone.0011021 PMC2882341

[56] Eo SK, Lee S, Chun S, Rouse BT (2001) Modulation of immunity against herpes simplex virus infection via mucosal genetic transfer of plasmid DNA encoding chemokines. J Virol 75: 569–578. 11134269 10.1128/JVI.75.2.569-578.2001 PMC113952

[57] Ramsay AJ, Husband AJ, Ramshaw IA, Bao S, Matthaei KI, et al (1994) The role of interleukin-6 in mucosal IgA antibody responses in vivo. Science 264: 561–563. 8160012 10.1126/science.8160012

[58] Beagley KW, Eldridge JH, Lee F, Kiyono H, Everson MP, et al (1989) Interleukins and IgA synthesis. Human and murine interleukin 6 induce high rate IgA secretion in IgA-committed B cells. J Exp Med 169: 2133–2148. 2786548 10.1084/jem.169.6.2133 PMC2189333

[59] Buck J, Ritter G, Dannecker L, Katta V, Cohen SL, et al (1990) Retinol is essential for growth of activated human B cells. J Exp Med 171: 1613–1624. 2332732 10.1084/jem.171.5.1613 PMC2187880

[60] Amaral CT, Pontes NN, Maciel BL, Bezerra HS, Triesta AN, et al (2013) Vitamin A deficiency alters airway resistance in children with acute upper respiratory infection. Pediatr Pulmonol 48: 481–489. 22833544 10.1002/ppul.22621 PMC7167945

[61] Lam HS, Chow CM, Poon WT, Lai CK, Chan KC, et al (2006) Risk of vitamin A toxicity from candy-like chewable vitamin supplements for children. Pediatrics 118: 820–824. 16882846 10.1542/peds.2006-0167

[62] Schuster GU, Kenyon NJ, Stephensen CB (2008) Vitamin A deficiency decreases and high dietary vitamin A increases disease severity in the mouse model of asthma. J Immunol 180: 1834–1842. 18209081 10.4049/jimmunol.180.3.1834

[63] de Francisco A, Chakraborty J, Chowdhury HR, Yunus M, Baqui AH, et al (1993) Acute toxicity of vitamin A given with vaccines in infancy. Lancet 342: 526–527. 8102669 10.1016/0140-6736(93)91648-6

[64] Hathcock JN, Hattan DG, Jenkins MY, McDonald JT, Sundaresan PR, et al (1990) Evaluation of vitamin A toxicity. Am J Clin Nutr 52: 183–202. 2197848 10.1093/ajcn/52.2.183

