# Supplementary figures and images for: Respiratory Tract Epithelial Cells Express Retinaldehyde Dehydrogenase ALDH1A and Enhance IgA Production by Stimulated B Cells in the Presence of Vitamin A
Source: PLoS One. 2014 Jan 22;9(1):e86554. doi: 10.1371/journal.pone.0086554 (PMC3899288; doi:10.1371/journal.pone.0086554)

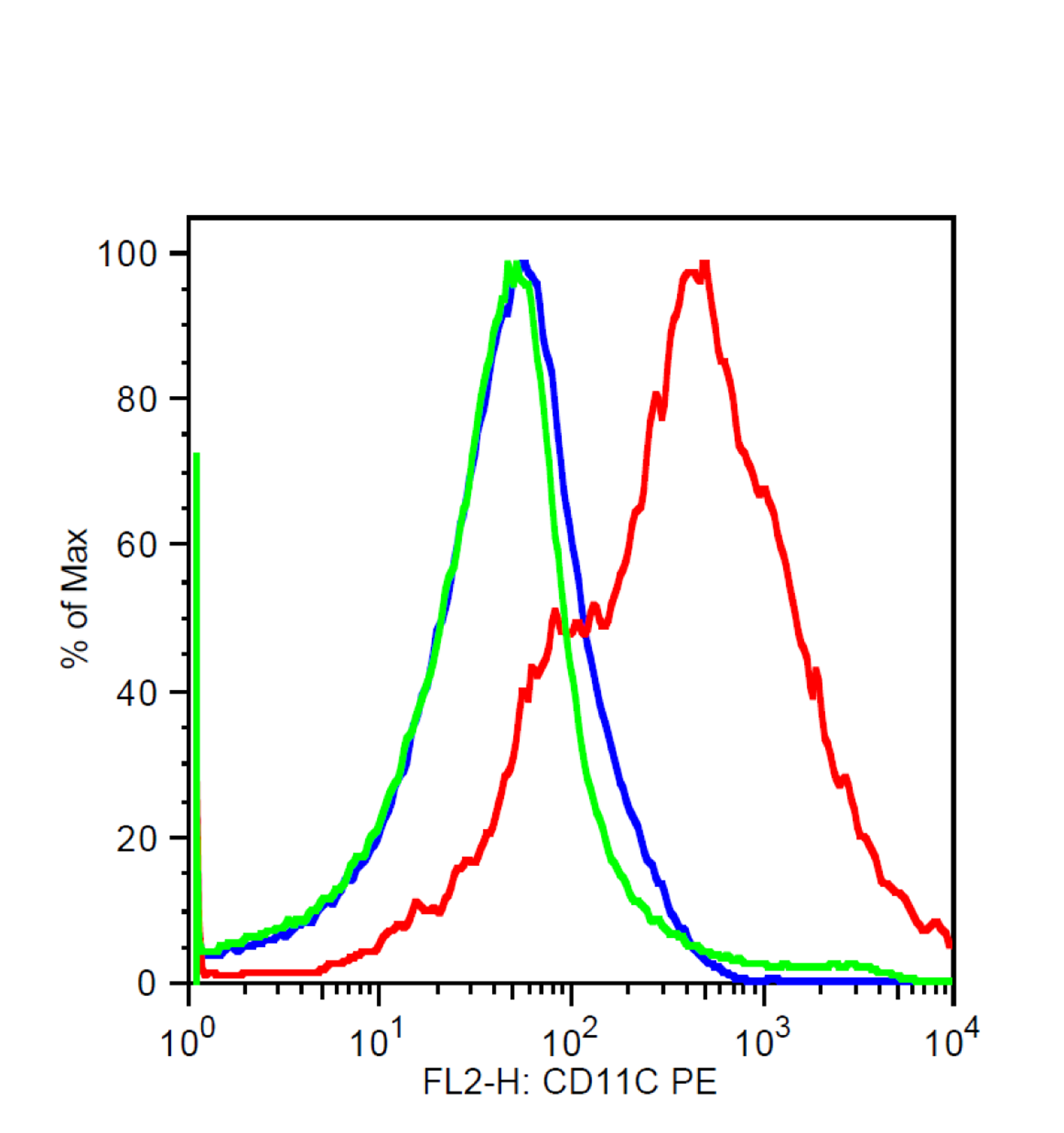

Supplement: Figure S1 — CD11c expression of selected NT cells. Following enrichment of CD11cHi and CD11cLo/neg cells from NT, phenotypes were confirmed by staining populations for membrane CD11c and examination by flow cytometry with a FACS Calibur. Live, non-RBC populations were first gated for analysis. Overlapping histograms represent the enriched CD11cHi cells (red), CD11cLo/neg cells (blue), and control naïve splenocytes (green). Relative cell numbers are shown on the Y axis and cell staining intensities are shown on the X axis. (TIF) [file pone.0086554.s001.tif]
